# Going Green or Going Away? A Spatial Empirical Examination of the Relationship between Environmental Regulations, Biased Technological Progress, and Green Total Factor Productivity

**DOI:** 10.3390/ijerph15091917

**Published:** 2018-09-03

**Authors:** Xueli Wang, Caizhi Sun, Song Wang, Zhixiong Zhang, Wei Zou

**Affiliations:** 1 Center for Studies of Marine Economy and Sustainable Development, Liaoning Normal University, 850 Huanghe Road, Dalian 116029, China; 376531665@163.com; 2 China Institute of Boundary and Ocean Studies, Wuhan University, Wuhan 430072, China; 3 Institute for the Development of Central China, Wuhan University, Wuhan 430072, China; SongW19911114@163.com; 4 College of Urban and Environment, Liaoning Normal University, 850 Huanghe Road, Dalian 116029, China; zhixiongzhang1991@foxmail.com (Z.Z.); zouwei_2003@sohu.com (W.Z.)

**Keywords:** biased technological progress, environmental regulation, green total factor productivity, spatial Durbin model

## Abstract

China’s economic development has resulted in significant resource consumption and environmental damage. However, technological progress is important for achieving coordinated economic development and environmental protection. Appropriate environmental regulation policies are also important. Although green total factor productivity, environmental regulations, and technological progress vary by location, few studies have been conducted from a spatial perspective. However, spatial spillover effects should be taken into consideration. This study used energy consumption, the sum of physical capital stock and ecological service value as total capital stock, the number of employed people as inputs, sulfur dioxide emissions as undesired outputs, and green GDP as total output to obtain green TFP through a slacks-based measure (SBM) global Malmquist-Luenberger Index. This study also estimated China’s biased technological progress under environmental constraints from 2004 to 2015 based on relevant data (e.g., green GDP, total capital stock, and employment figures). The relationship between green total factor productivity (GTFP), technological progress, and environmental regulation was then examined using a spatial Durbin model. Results were as follows: (1) Based on the complementary elements, although the labor costs gradually increase, the rapid accumulation of capital leads to technological progress that is biased toward capital. However, technological progress in the labor bias can significantly increase GTFP. (2) There is a u-shaped relationship between existing environmental regulations and GTFP. Technological progress can significantly promote GTFP in the surrounding areas through existing environmental regulations. (3) Under spatial weight, the secondary industry coefficient was negative while human capital stock and FDID had positive effects on GTFP. Technological progress is the source of economic growth. It is therefore necessary to promote biased technological development and improve labor-force skills while implementing effective environmental regulation policies.

## 1. Introduction

China has experienced rapid economic growth over the past 40 years as a result of economic reforms. It is now the world’s second largest economy. China’s economic achievements have attracted worldwide attention. However, these achievements are mainly the result of heavy energy and resource consumption. China’s energy consumption increased more than four times between 2000 and 2008 when compared with data from the 1990s [1]. Significant energy consumption and low-tech industries have resulted in increased environmental pressure. Even in the more developed eastern provinces, many companies still produce and provide low value, energy-inefficient, and environmentally harmful products. This trend has also spread to the central and western regions. If such development cannot be altered and improved, Chinese companies will lack sustainable profitability, thus becoming dependent on limited resource and environmental destruction in exchange for economic growth [2]. Although this type of economic growth can improve the quality of life for many people, it has caused serious environmental pollution. This has forced China to construct a resource-saving, environmentally friendly society by developing a large number of costly environmental regulations.

In consideration of resources and environmental restrictions, the contradiction between environmental and social economic development is increasing in both amount and structure. Public attention to environmental quality is also growing. Green GDP and green productivity are the new demands for current economic growth; they also form the basis of China’s economic transformation. China first proposed a sustainable national development strategy in 1996. The “13th Five-Year Plan” proposed to accelerate an improved ecological environment. The 2014 Global Environmental Performance Index Assessment Report ranked China 118 of 180 countries. In 2018, China ranked 120. This is evidence that China’s environmental friendliness is unsatisfactory compared to other developed countries. It is clear that China’s rapid economic development has resulted in pressure on the local environment.

In sustainable development, resources and the environment are not only endogenous variables of economic development, but also rigid constraints on the scale and speed of economic development. One of the main reasons why some regions in China have embarked on an extensive and unsustainable development path of high investment and high output is that the main indicator for traditionally evaluating the economic performance of a region is the GDP, which only considers output. The constraints of input are not considered as an important engine of economic growth, that is, productivity growth.

In order to incorporate input constraints into the framework of assessing economic performance, economists have proposed a measure of total factor productivity. In the same vein, they have also proposed to measure the economic performance of a region with total factor productivity. This will not only reveal the overall quality of China’s economic growth, but also reflect the environmental factors indicating whether economic growth is sustainable. However, the traditional total factor productivity only considers the input constraints of production factors, such as labor and capital, and does not consider the constraints of the resource environment. It distorts the evaluation of social welfare changes and economic performance, thus misleading policy recommendations [3].

Under conditions of energy consumption and environmental pollution, this study analyzed the factors influencing China’s green total factor productivity (GTFP) and calculated its additive Luenberger index. Facing incompatibility between environmental protection and economic growth, China’s environmental regulations and technological progress have become important breakthroughs. At the same time, using technological progress to measure innovation will help promote both environmental protection and economic growth [4]. This may solve China’s complex problems, in which “innovation capacity is not strong enough”, “the level of the real economy needs to be improved”, and “eco-environment protection is a long and arduous task” [5].

## 2. Literature Review

In the calculation of total factor productivity, the traditional measure of total factor productivity (such as Fischer index and Törnqvist index) cannot be accounted for under the productivity of resource and environmental constraints. The traditional distance function Malmquist productivity index does not need to consider price information, but cannot consider the total factor productivity in the presence of “bad” output. When there is excessive input or insufficient output, the existence of slack variables at this time will make the research highly evaluate the efficiency of the object, while the data envelopment analysis (DEA) efficiency measure of the angle ignores the input or output aspect. Hence, the calculated efficiency result is not accurate. To overcome these two shortcomings, Färe and Grosskopf [6] and Fukuyama and Weber [7] further developed Tone’s [8] non-radial, non-angle slack-based measure (SBM) efficiency measure. Generalized non-radial, non-angular directional distance function. In order to adapt to the non-angular, directional distance function with additive structure, Chambers et al. [9] proposed a productivity measure with additive structure, the Luenberger productivity indicator.

The existing research has undertaken a positive exploration of the interface between environmental regulation and green development. However, mixed results have been achieved. Zhang and Wei [10] discussed the nonlinear relationship between environmental regulations and carbon emissions, and pointed out that, when the intensity of environmental regulations shifts from weak to strong, the “green paradox” effect becomes a “reverse reduction” effect. However, environmental regulations make foreign direct investment (FDI) appear as a “pollution refuge” effect. A study by Yuchanglin et al. [11] analyzed the influence of environmental regulations on Chinese environmental pollution from the perspective of the invisible economy. Its results indicated that the higher the intensity of environmental regulations, the greater the negative impact of the invisible economy on China’s environment. Yun-So and Liu [12] assert that environmental regulations can suppress regional economic development by increasing enterprise production costs and promoting technological progress. Most scholars believe that governments should embrace technological progress and the associated biases while gradually increasing environmental protections and reducing emissions. Current international research on biased technological progress is mostly based on a study by Acemoglu [13], which demonstrated that technological progress is affected by both the factor price and scale effects. Most research on Chinese biased technology is based on the Chinese Technology Progressive Index defined by Dai and Xu [14]. The results indicated that China’s technological progress between 1978 and 2005 was primarily biased toward capital, which is an increasing tendency. Jing and Zhang [15] believe that reasonable environmental regulations can alter the current direction of technology and help Chinese industries integrate green technological development. Song and Wang [16] measured biased technological progress using the DEA method. They divided technological progress into environmental-biased and pollution-biased. Their results indicated that environmental-biased technological progress can promote environmental friendliness.

Previous research has primarily examined the influence of environmental regulations on production efficiency, technological innovation, industrial optimization, FDIs, and opening-up. This provides a rich theoretical basis for our study. The representative theories are as follows.

The first involves “follow cost”, which is the traditional neo-classic belief that strict environmental regulation will introduce the negative externalities of pollution into enterprise production costs, thus reducing production efficiency and profit. Production scale adjustments, resource allocations, and other behaviors also affect the industrial structure through enterprise entry or exit [17]. The second is the “pollution refuge hypothesis”, which argues that, in order to avoid restrictions or reduce compliance costs in an open economy, international differences in environmental standards or regulatory levels in terms of trade or investment will lead to cross-border transfers of polluting industries [18,19,20]. This will result in adjustments to the national or regional industrial structure. The third is the “Porter hypothesis”, which posits that strict and appropriate environmental regulations will inspire enterprises to change their production processes, guide them to actively seek improved resource utilization efficiency, stimulate technological innovation to reduce environmental compliance costs, produce an innovative compensation effect, and achieve the Pareto improvement [21,22]. There are academic discrepancies regarding whether regulation intensity should be greater and environmental standards should be stricter to reduce pollution emissions. Xu [23] questioned the Porter hypothesis by asserting that it must be based on the “proper design of environmental regulation”. A study by Jin [24] indicated that costly environmental regulations will eliminate many companies. There is a limit to the number of companies that can be eliminated in a country or region within a certain period of time. Environmental regulations are unsustainable if this limit is exceeded.

In the study of environmental regulation and green total factor productivity, most studies focus on industry research and efficiency evaluation, with relatively few applications in regional economic research. For example, Jiang et al. [25] examine the spillover effects of FDI on green technology progress rate (as measured by the green total factor productivity), and found that regardless of the industry under the high and low environmental regulations, the labor-based FDI has a negative spillover effect. The FDI of capital has a positive spillover effect. Research by Zhijun [26] found that environmental regulation and green innovation can promote environmental planning, thereby promoting the green transformation of industrial growth. Yantuan [27] and others calculated eco-efficiency using the Meta-US–SBM method, and analyzed the differences in spatial and temporal ecological efficiency. The results indicate that the ecological efficiency in eastern China was higher than that in the central and western regions.

Whether the current environmental regulation policy will exert greater pressure on China’s future growth depends on the development of GTFP. Therefore, this study attempted to discover whether China’s GTFP would continue to grow under current environmental regulations. Technological progress as a measure of productivity is closely linked to China’s green economic development. The existing research has done extensive work on environmental regulation, green total factor productivity, and biased technology advancement. However, there are few existing attempts to combine the three, especially when studying green total factor productivity. This study thus attempted to expand the existing literature in the following ways:

1. Technological progress is directional. This study therefore used the standardized supply surface system method to calculate biased technological progress. This method is very reliable. The algorithm also considers the sufficiency and insufficiency of elements under environmental constraints and can be used to obtain the technological progress deviation more accurately while factors are abundant and insufficient.
2. The Porter hypothesis suggests that appropriate environmental regulations will promote enterprise innovations. This study adds the product of technological progress and environmental regulation to the model to explore the effect of environmental regulation on green total factor productivity under the influence of technological advancement.
3. Technological progress has a spatial diffusion effect, and the formulation of environmental regulation policies in various provinces and cities will also be affected by neighboring provinces. The spatial Durbin model can examine the influence of the dependent variables affected by the variables in the local area, as well as the dependent and independent variables in neighboring areas. Therefore, this paper will focus on the spatial relationship between technological progress, environmental regulation, and green total factor productivity.

## 3. Methodology and Data

### 3.1. Green Total Factor Productivity (GTFP) Measurement

This study selected the additive Luenberger index proposed by Fukuyama and Weber [7] to measure China’s GTFP. The Luenberger index is based on relaxation of the directional distance SBM function [9]. The SBM-based global directional distance function and the global Luenberger index were constructed based on the global Malmquist-Luenberger index proposed by Oh [28]. In conjunction with cyclic accumulation, the global Luenberger index can not only be used to analyze short-term changes in total factor productivity, but also observe long-term trends.

#### 3.1.1. SBM Directional Distance Function

The specific form of the SBM directional distance function is as follows:

(1) $$\begin{array}{c} S^{G,k^{\prime }}\left(x^{t,k^{\prime }},y^{t,k^{\prime }},b^{t,k^{\prime }},g^{x},g^{y},g^{b}\right)\\ \;=\begin{array}{c} max\\ S_{x},S_{y},S_{b} \end{array}\frac{1}{2N}\overset{N}{\underset{n=1}{\sum }}\frac{S_{n,x}^{G,k^{\prime }}}{x_{n}^{t,k^{\prime }}}+\frac{1}{M+1}\left[\overset{M}{\underset{m=1}{\sum }}\frac{S_{m,y}^{G,k^{\prime }}}{y_{m}^{t,k^{\prime }}}+\overset{I}{\underset{i=1}{\sum }}\frac{S_{i,b}^{G,k^{\prime }}}{b_{i}^{t,k^{\prime }}}\right] \end{array}$$

(2) $$s.t.\overset{T}{\underset{t=1\;}{\sum }}\overset{K}{\underset{k=1}{\sum }}z^{t,k}x_{n}^{t,k}+S_{n,x}^{G,k^{\prime }}=x_{n}^{t,k^{\prime }};$$

(3) $$\overset{T}{\underset{t=1\;}{\sum }}\overset{K}{\underset{k=1}{\sum }}z^{t,k}y_{m}^{t,k}-S_{m,y}^{G,k^{\prime }}=y_{m}^{t,k^{\prime }};$$

(4) $$\overset{T}{\underset{t=1\;}{\sum }}\overset{K}{\underset{k=1}{\sum }}z^{t,k}b_{i}^{t,k}-S_{i,b}^{G,k^{\prime }}=b_{i}^{t,k^{\prime }}$$

(5) $$z^{t,k}\geq 0;S_{n,x}^{G,k^{\prime }}\geq 0;S_{m,y}^{G,k^{\prime }}\geq 0;S_{i,b}^{G,k^{\prime }}\geq 0;n=1\cdots \cdots N,m=1\cdots \cdots M,i=1\cdots \cdots I$$

where $S^{G,k^{\prime }}$ represents the distance of decision unit $k^{\prime }$ from the global production frontier. The measured distance is effectively defined as the direction of $g=\left(-x^{t},y^{t},-b^{t}\right)$, which minimizes input, increases the desirable output, and reduces the undesirable output. When it is at 0, the decision-making unit (DMU) is located on the production frontier and there is no technical inefficiency. $\left(x^{t,k^{\prime }},y^{t,k^{\prime }},b^{t,k^{\prime }}\right)$ is the input and output vector of the province *k’*. $\left(g^{x},g^{y},g^{b}\right)$ is the direction vector, representing the reduced inputs, increased desirable outputs, and reduced undesirable outputs. $\left(S_{n,x}^{G,k^{\prime }},S_{m,y}^{G,k^{\prime }},\;S_{i,b}^{G,k^{\prime }}\right)$ represents the slack variables, reflecting the degree of overcapacity of inputs, reduced desirable outputs, and undesirable outputs.

**Figure 1:**
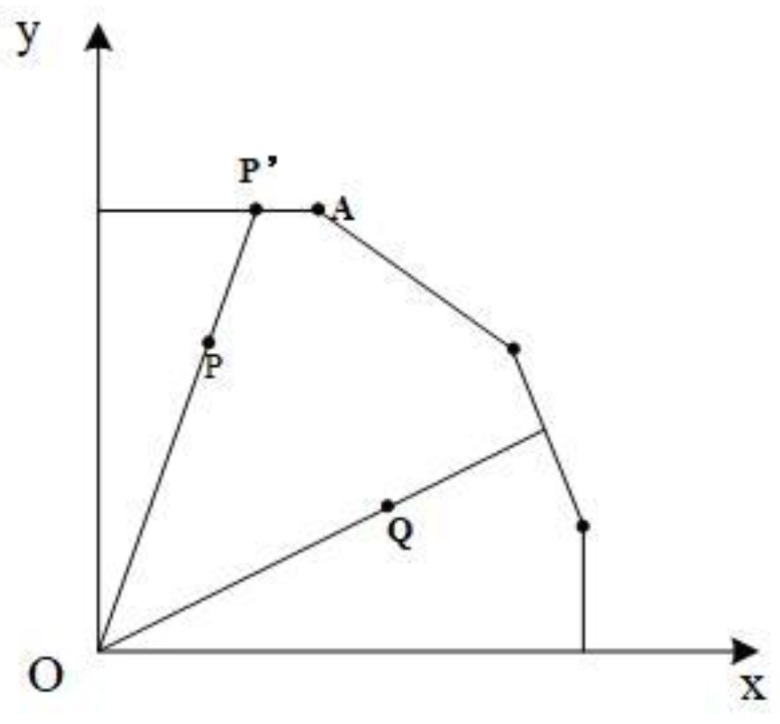
Slack variable schematic.

The frontier of the DEA model is composed of piecewise linear functions. The piecewise linear function will appear parallel to the coordinate axis in the space coordinate system, which is the source of the slack problem. In Figure 1, we take a single output and a single input as an example. P’ is the projection of P on the frontier, but P’ is insufficiently produced (i.e., P’A segment) compared to point A on the frontier. If $x_{t}-X\alpha =0$ or $y_{t}-Y\alpha =0$, there is no input slack variable or output slack variable. In the linear programming of radial DEA, the constraints are expressed in the form of inequalities, rather than equations, which are the basis for the existence of slack variables.

The significance of the slack variable is that the observation point is best when each of its elements are at 0, otherwise there are still areas to be improved. When $S_{n,x}^{G,k^{\prime }}$, $S_{m,y}^{G,k^{\prime }}$, and $S_{i,b}^{G,k^{\prime }}$ are all greater than 0, the actual desirable output is less than the desirable output of the frontier boundary. Moreover, both the actual inputs and undesirable outputs are greater than the inputs and undesirable outputs of the frontier boundary. The slack variable can measure the deviation of the best distance from the observation point, so the larger the $S_{n,x}^{G,k^{\prime }}$, $S_{m,y}^{G,k^{\prime }}$, and $S_{i,b}^{G,k^{\prime }}$ are, the greater the amount of input, the desirable output, and the excess output produced by the undesirable outputs too [29].

#### 3.1.2. DDF and GML Productivity Index

Färe et al. [30] developed the Malmquist productivity index proposed by Caves et al. (1982) [31] and applied it to a variety of research fields. In 1997, Chung et al. constructed the directional distance function according to the Luenberger [32] shortage function idea, which can solve the problem of evaluating the efficiency of undesired output. This article considers environmental factors in this model. The definition is as follows:

(6) $$\vec{D}\left(x,y,b;\;\vec{g}\right)=\sup\left\{\beta :\left(y,b\right)+\beta \vec{g}\in P\left(x\right)\right\}$$

In the above equation, x is the input vector, y and g are the desired output vectors, and b and g are undesired output vector. $\vec{g}$ = ($\vec{g_{x}},\vec{g_{y}},\;\vec{g_{b}})$ is the direction vector, which can be configured to result in a simultaneous increase in desirable outputs and a decrease in undesirable outputs. This article assumes g=(y, −b), represented in the case where, for a given input *x*, the desired output and undesirable output expand and reduce proportionally. β is the maximum possible value for the expected output of y growth and the undesired output of b reduction. Suppose each DMU uses *N* inputs $x=\left(x1,\ldots ,xN\right)\in R_{+}^{N}$ to obtain *M* kinds of expected outputs $y=\left(y1,\ldots ,yM\right)\in R_{+}^{M}$ and *I* kinds of undesired outputs $b=\left(b1,\ldots ,bI\right)\in R_{+}^{I}$. Then, $P^{t}\left(x\right)$ denotes the set of production possibilities for the *t* = 1, …, T period: $P^{t}\left(x\right)=\left\{\left(y^{t},b^{t}\right):\;x^{t}\to \left(y^{t},b^{t}\right)\right\},\;x\in R_{+}^{N}$.

To enhance the comparability of the technical efficiency of the DMUs, Oh [8] defines the global production technology set as the union of all current production technology sets, $P^{G}\left(x\right)=P^{1}\left(x^{1}\right)\cup P^{2}\left(x^{2}\right)\cup \ldots \cup P^{T}\left(x^{T}\right)$. Under the single production front, the calculated GTFP is comparable between each DMU and each time period. Therefore, the GTFP defined according to the global production technology set and the directional environmental distance function has certain advantages.

Given the DDF defined above and the global technology set, the new index, namely, the Global Malmquist Luenberger (GML) productivity index, is circular and free from the other problems inherent in the ML index. The GML index can readily be specified as:

(7) $$GML_{t}^{t+1}=\frac{1+{\vec{D}}^{G}\left(x^{t},y^{t},b^{t};y^{t},b^{t}\right)}{1+{\vec{D}}^{G}(x^{t+1},y^{t+1},b^{t+1};y^{t+1},b^{t+1}}$$

In the above equation, *t*, *t* + 1 refer to time periods, while ${\vec{D}}^{G}$ is the distance function defined on the global technology set.

Previous studies were typically based on the measurement principle, which decomposes the change of total factor productivity into technological change ($TC_{t}^{t+1}$) and efficiency change $\left(EC_{t}^{t+1}\right)$. This paper measures the sum of the two. The is because, on one hand, the efficiency of our measurements is a relative concept that is actually measured by TFP changes. These changes may be because of a better combination of existing elements and production, and not necessarily related to the introduction of new production factors or technological innovations. On the other hand, changes in efficiency reflect the ability of provinces and regions to absorb existing knowledge, technology, and production elements. Efficiency change should be a key variable in the study of economic development. From these two perspectives, the overall productivity index is a combination of usual technological and efficiency changes. It embodies the continuous catch-up process of each DMU to the global front. This is a relatively reasonable measurement method.

### 3.2. Biased Technical Progress Measurement

#### 3.2.1. The Direction of Technical Progress

The production function should be set before measuring the direction of China’s technological progress. Technological progress cannot affect the marginal output ratio of capital and labor in the traditional Cobb–Douglas production function. Therefore, the traditional Cobb-Douglas production function cannot be used to study the direction of technological progress. This article refers to David and Kluner’s [33] research by adopting the CES production function to study biased technological progress:

(8) $$Y_{t}={\left[\left(1-\alpha \right){\left(A_{t}L_{t}\right)}^{\frac{e-1}{e}}+\alpha {\left(B_{t}K_{t}\right)}^{\frac{e-1}{e}}\right]}^{\frac{e-1}{e}}$$

Here, $Y_{t}$ is the output in period *t*. $L_{t}$ and $K_{t}$ are the inputs of labor and capital, respectively. α is capital intensity, and e∈(0, +∞) is the alternative elasticity of labor and capital. $A_{t}$ and $B_{t}$ are labor efficiency and capital efficiency, respectively (also known as labor-enhanced technological progress and capital-enhanced technological progress, respectively).

According to Acemoglu [13], if technological progress leads to an increase (decrease) in the capital output ratio, technological progress is biased toward capital (labor). If it does not affect the capital output ratio, then technological progress is neutral. That is, if $\frac{\partial ∆}{\partial B/A}\frac{d\left(B/A\right)}{dt}$ is greater than 0, technological progress is biased toward capital; if it equals 0, technological progress is neutral; if less than 0, technological progress is labor-biased.

This definition clarifies the deviation of technological progress. In addition, we have quantitatively studied the degree of bias in technological progress to obtain more information. This study used the technical progress direction index ($D_{t}$) designed by Dai and Xu [14] to estimate technologically biased progress under environmental constrains, as follows:

(9) $$D_{t}=\frac{1}{∆}\frac{\partial ∆}{\partial B/A}\frac{d\left(B/A\right)}{dt}=\frac{e-1}{e}\left(\frac{A_{t}}{B_{t}}\right)\frac{d\left(B_{t}/A_{t}\right)}{dt}$$

$D_{t}$ is the biased technology progress index, which is mainly determined by the change of substitution elasticity e and $\frac{B_{t}}{A_{t}}$. When e < 1, there is a complementary relationship between capital and labor. If $\frac{B_{t}}{A_{t}}$ rises (decreases), technological progress is biased toward labor (capital), and the direction of technological progress and capital output ratio change in the opposite direction. When e > 1, there is an alternative relationship between capital and labor. If $\frac{B_{t}}{A_{t}}$ rises (decreases), technological progress favors capital (labor), and the direction of technological progress and capital output ratio positively change. When e = 1, technological progress is neutral. In the final measurement results, $D_{t}>0$ indicates that technological progress is biased toward capital, while $D_{t}<0$ indicates that technological progress is biased toward labor.

The environmental issue has increased the actual cost of China’s economic growth. Considering the environmental impact, the technology bias may change. To estimate the index of technological progress under China’s environmental constraints, this study estimated the elasticity of substitution e, capital-enhanced technological progress $B_{t}$, and labor-enhanced technological progress $A_{t}$.

#### 3.2.2. Estimating Capital-Enhanced Technological Progress and Labor-Enhanced Technological Progress

By assuming that the price of capital and labor is its marginal output and bringing it to (1), we can obtain capital-enhanced technological progress $B_{t}$ and labor-enhanced technological advance $A_{t}$:

(10) $$A_{t}=\frac{Y_{t}}{L_{t}}{\left(\frac{w_{t}L_{t}}{\left(1-\alpha \right)\left(w_{t}L_{t}+r_{t}K_{t}\right)}\right)}^{\frac{e}{e-1}}$$

(11) $$B_{t}=\frac{Y_{t}}{K_{t}}{\left(\frac{r_{t}K_{t}}{\alpha \left(w_{t}L_{t}+r_{t}K_{t}\right)}\right)}^{\frac{e}{e-1}}$$

$w_{t}$ and $r_{t}$ are the wage rate and capital rent rate, respectively, while α is capital intensity.

#### 3.2.3. Alternative Elasticity and Capital Intensity Estimation

With the further development of alternative elasticity, the CES and VES production functions are used to estimate substitution elasticity. Considering the technological progress bias, we chose the CES production function to estimate substitution elasticity. Klump et al. [34] proposed the efficacy of the “standardized supply surface system method”, which has been widely used in empirical research. This study referred to research by Leon-Ledema et al. [35], Dai and Xu [14], and Chen and Lian [36]. Based on reasonable simplification of the “Box–Cox” factor efficiency growth rate, we standardized the variables themselves using the sample mean of each variable to obtain a standardized system. The equations are as follows:

(12) $$log\left(\frac{Y_{t}}{\bar{Y}}\right)=log\left(\xi \right)+\frac{e}{e-1}log\left\{\left(1-\alpha \right){\left[\frac{L_{t}}{\bar{L}}exp\left[\bar{t}\frac{\gamma_{L}}{\lambda_{L}}\left({\left(\frac{t}{\bar{t}}\right)}^{\lambda_{L}}-1\right)\right]\right]}^{\frac{e-1}{e}}+\alpha {\left[\frac{K_{t}}{\bar{K}}exp\left[\bar{t}\frac{\gamma_{k}}{\lambda_{k}}\left({\left(\frac{t}{\bar{t}}\right)}^{\lambda_{k}}-1\right)\right]\right]}^{\frac{e-1}{e}}\right\}$$

(13) $$log\left(\frac{w_{t}L_{t}\;}{Y_{t}}\right)=log\left(1-\alpha \right)+\frac{e-1}{e}log\left(\xi \right)-\frac{e-1}{e}log\left(\frac{\frac{Y_{t}}{\bar{Y}}}{\frac{L_{t}}{\bar{L}}}\right)+\frac{e-1}{e}\bar{t}\frac{\gamma_{L}}{\lambda_{L}}\left[{\left(\frac{t}{\bar{t}}\right)}^{\lambda_{L}}-1\right]$$

(14) $$log\left(\frac{r_{t}K_{t}}{Y_{t}}\right)=log\left(\alpha \right)+\frac{e-1}{e}log\left(\xi \right)-\frac{e-1}{e}log\left(\frac{\frac{Y_{t}}{\bar{Y}}}{\frac{K_{t}}{\bar{K}}}\right)+\frac{e-1}{e}\bar{t}\frac{\gamma_{k}}{\lambda_{k}}\left[{\left(\frac{t}{\bar{t}}\right)}^{\lambda_{k}}-1\right]$$

Among them, $\bar{Y},\;\bar{L}$, $\bar{K}$ are the geometric means of output, labor and capital, respectively. $\bar{t}$ is the arithmetic mean of the year. ξ is the scale factor introduced for standardization. $\gamma_{L}$ and $\gamma_{k}$ are the technical growth parameters of labor and capital, respectively, while $\lambda_{L}$ and $\lambda_{k}$ are technical curvatures.

The annual output $Y_{t}$, labor input $L_{t}$, capital input $K_{t}$, labor income $w_{t}L_{t}$, and capital income $r_{t}K_{t}$ were used to estimate the system equation, alternative elasticity e, and capital intensity α in each province.

### 3.3. Environmental Regulations

Böcher [37] categorized broad environmental regulations into various types (e.g., persuasive, cooperative, economical, and controlling). This is because economic regulations can realize the external cost internalization more effectively according to the developmental reform trend. This article therefore discusses economic environmental regulations. Considering data availability, economic environmental regulations can be further divided into “economic incentives” (e.g., sewage charge income) and “governance input” (i.e., the total investment in treating environmental pollution). Obtaining the total amount of governmental pollution investment involves measuring the cost of pollution control not related to economic characteristics. In this study, we chose the sewage charge as the environmental regulation variable. It is expressed by $ER_{it}$, measured in constant prices for the year 2000, and reduced with the GDP deflator.

### 3.4. Spatial Durbin Model

To test the interaction of regions empirically, previous studies, such as Fredriksson and Millimet [38], Konisky [39], and Li et al. [40] focused on the spatial autoregressive coefficient ρ in the following equation.

(15) $$Y_{it}=\alpha +\rho \overset{N}{\underset{j=1,i\neq j}{\sum }}W_{ij}Y_{jt}+\beta X_{it}+\mu_{i}+\varphi_{i}+\varepsilon_{it}$$

In order to obtain consistent and accurate estimates, some issues should be addressed here. The first is $\sum_{j=1,i\neq j}^{N}W_{ij}Y_{jt}$ may have endogeneity. The dependent variable contains $Y_{it}$ and both functions. The ordinary least squares (OLS)estimates will be biased because of the above simultaneity problem. In order to solve endogeneity, Konisky [38] used the instrumental variable approach. However, it is difficult to find suitable instruments (Zhang et al. [41]). Noting the instrumental variable approach may lead to inaccurate estimates, Anseilin [42] proposed using maximum likelihood estimation to address this endogeneity problem. Following LeSage and Pace [43,44] and Zhang [41], this paper employs the ML (maximum likelihood) approach to estimate the spatial econometric model.

The second issue is that there is a significant correlation between technological diffusion and geographical distance [45]. Pollution spillover reflects interregional externalities and implies distortion. The pollution source reduces the local environmental pollution level by transmitting pollutants to outside areas. This behavior reduces the opportunity cost of environmental regulations and enhances the competitive advantage of local high-pollution activities. However, in areas where pollution has been transferred, the level of environmental pollution has increased. Thus, the competitive advantage of high-pollution activities may decline. Pollution spillover is also a key factor in the strategic behavior involved in regional environmental regulation.

This paper mainly investigates the role and mechanism of environmental regulation and biased technological progress on the impact of green total factor productivity. Considering that the spatial Durbin model (SDM) can examine the influence of the dependent variables affected by the variables in the local area, as well as the dependent and independent variables in neighboring areas, this makes the SDM more suitable for the purposes of this paper.

The relationship between technological progress, environmental regulations, and GTFP was further examined after calculating the bias index for technological progress. To ensure that the regression was robust, we introduced FDID, human capital stock, and industrial structure as control variables in addition to constructing the following model [46]:

(16) $$Y_{it}=\rho \overset{N}{\underset{j=1}{\sum }}W_{ij}Y_{jt}+\beta X_{it}+\overset{N}{\underset{j=1}{\sum }}\theta W_{ij}X_{jt}+\mu_{i}+\varphi_{i}+\varepsilon_{it}$$

Based on the traditional spatial Durbin model, when *θ* is 0, the SDM model can be transformed into a spatial lag model. When θ+ρβ is 0, the SDM model can be transformed into a spatial error model. $\varphi_{i}$ indicates the time fixed-effect, $\mu_{i}$ indicates the individual fixed-effect, and $\varepsilon_{it}$ indicates the random disturbance term. There may be a nonlinear relationship between environmental regulations and green development. Technological progress will also be affected by existing environmental regulations. This study therefore added the square term of environmental regulations to the model to analyze its nonlinear relationship while simultaneously adding the interactions between environmental regulations and technological progress to analyze the moderating effect. The model is as follows:

(17) $$\begin{array}{l} GTFP_{it}=\alpha_{1}+\rho W\times GTFP_{it}+\alpha_{2}tec_{it}+\alpha_{3}ER_{it}+\alpha_{4}indus_{it}+\alpha_{5}FDI_{it}+\alpha_{6}educ_{it}\\ \;+\alpha_{7}tec_{it\;}\times ER_{it}+\alpha_{8}ER^{2}{}_{it}+\alpha_{9}W\times tec_{it}+\alpha_{10}W\times ER_{it}\\ \;+\alpha_{11}W\times indus_{it}+\alpha_{12}W\times FDI_{it}+\alpha_{13}W\times educ_{it}\\ \;+\alpha_{14}W\times \left(tec_{it}\times ER_{it}\right)+\alpha_{15}W\times ER^{2}{}_{it}+\varepsilon_{it} \end{array}$$

$Y_{it}$ is the GTFP and W is the *N* × *N*-order social and spatial weight matrix. Environmental regulations not only affect the local area, but also the surroundings. This is also true for technological progress. We used the economic and social distance spatial weight matrix when considering the relationship between GTFP, environmental regulations, and biased technological progress. $W\times X_{it}$ indicates the spatial effect of different variables. ρ is a spatially delayed regression coefficient. $tec_{it}$ represents biased technological progress, and $ER_{it}$ represents environmental regulations. The controls are human capital stock $educ_{it}$, and $FDID_{it}$ is foreign trade dependence. Secondary industries occupy a relatively high proportion of the total industry in most provinces. Such industrial development relies on resource consumption. This study attempted to discuss China’s green development. Thus, the industrial structure was measured by assessing the proportion of second industry. In order to further study the impact of the existing degree of China’s environmental regulation on green development, we attempted to join the square of environmental regulation $ER^{2}{}_{it}$ in order to find out whether there is a nonlinear relationship between environmental regulation and green economy development. We introduced the interaction between technological progress and environmental regulations as $tec_{it}\times ER_{it}$. This enabled further study of the impact of technological progress on GTFP under existing environmental regulations.

### 3.5. Data and Variable Selection

The indicators involved in measuring GTFP are green GDP, capital stock, labor, energy consumption, and sulfur dioxide emissions. We used labor input, capital investment, labor income, capital income, and total output when computing biased technological progress. The indicator selection and data sources are as follows:

$Y_{t}$ is the total output. We adopted green GDP to represent China’s total output when considering environmental constraints. Green GDP represents the level of economic development in each region, for which we used the year 2000 as the base period for deflation. We then used GDP minus the depreciation of fixed assets and the cost of environmental protection to obtain green GDP. The cost of environmental protection includes environmental protection inputs, waste gas, and waste water multiplied by unit value.

$w_{t}L_{t}$ and $r_{t}K_{t}$ represent labor income and capital income, respectively. We referred to research by Dai and Xu (2010) [14] when dividing the net production tax into labor income and capital income.

$K_{t}$ represents capital output when calculating technological progress and capital stock for measuring GTFP. We used the sum of the regional ecological service value (i.e., 100 million yuan) and regional capital stocks (i.e., 100 million yuan) to represent regional capital output. Referring to the global ecosystem service framework constructed by Costanza [47] and Sun’s [48] related research methods, we used the market value, expert estimation, transfer payment, and system engineering methods to measure the supply service, environmental adjustment, social and human values, and support service values of all provinces and cities. This sum indicates total ecological services value. Capital stock has been extended to 2015 based on Shan [49], while deflation was carried out using 2000 as the base year. We obtained total capital stock using the sum of the above two calculations.

$L_{t}$ is labor input (i.e., regional year-end labor employment).

$FDID_{t}$ is yearly foreign trade dependence. This is measured using the ratio of yearly actual FDI use to real GDP.

$Educ_{it}$ is human capital as referred to by the “China Human Capital Report”.

$indus_{it}$ is the proportion of second industries.

The current research objective did not include an empirical analysis of Tibet, Hong Kong, Macao, or Taiwan because of the special endowment and data availability. Relevant data from 2003 to 2015 was selected using the China Statistical Yearbook, China Demographic Yearbook, China Environment Yearbook, and the New China 60 Statistical data compilation. Descriptive statistics of the data are shown in Table 1.

## 4. Results Analysis

### 4.1. Analysis of GTFP Spatiotemporal Disparity

**Figure 2:**
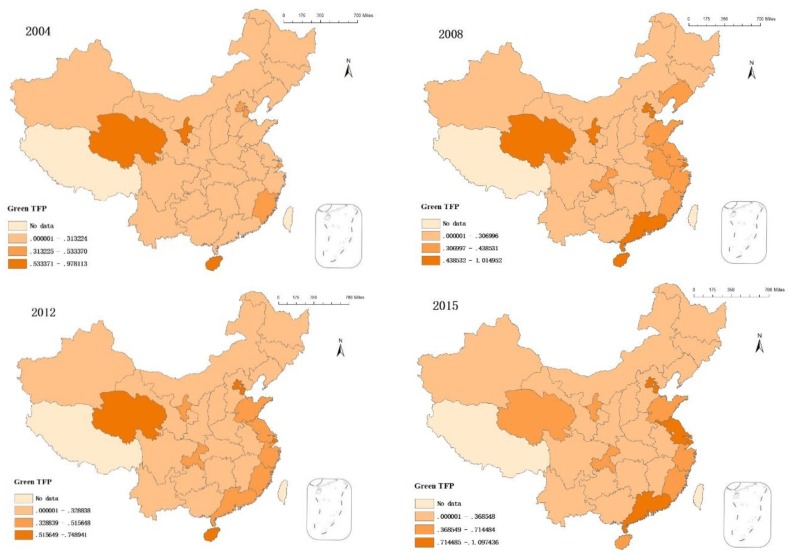
The temporal and spatial evolution of GTFP.

This study used the MaxDEA Pro software to calculate GTFP in 30 Chinese provinces and cities according to the super-efficiency SBM–Global–Luenberger formula. This study divided the nationwide 2004–2015 GTFP into three levels using the ArcGIS natural breakpoint classification method. Regional average value was calculated to analyze temporal and spatial evolution (Figure 2).

GTFP has increased in various Chinese provinces and cities from 2004 to 2008. There was a significant increase in the number of second and third-level cities during this period. This indicates improved green development. China implemented its Eleventh Five-Year Plan in 2005 to construct a resource-saving and environment-friendly society. National policy is focused on developing a circular economy. There have been proposals to protect and restore the natural ecology and increase environmental protection. The number of third-level cities decreased between 2008 and 2012; their values also declined. This indicates that China’s GTFP slowed during that period. This assertion is based on research by Dong [3], who studied China’s environmental quality index. The index dropped significantly during this period while reduced GTFP was also connected to declining environmental quality. The number of third-level provinces and cities increased between 2012 and 2015. This occurred mostly in the eastern region. Thus, there have been some achievements arising from the coordinated development of China’s green economy, while gradual economic developments and transformations have occurred in some of the developed eastern areas.

From a spatial perspective prior to 2012, third-level cities were mostly high ecological service value areas (e.g., Qinghai, Ningxia, and Hainan). These areas have certain ecological and environmental bases. Local industries are relatively underdeveloped, but there are environmental advantages. Therefore, GTFP is relatively high. However, GTFP value in the eastern coastal areas increased after 2008. This indicates that some of the developed provinces and cities will gradually realize green development under existing environmental protections and economic development. Beijing, Tianjin, Jiangsu, Shanghai, and Guangdong were considered third-level cities as of 2015. This indicates that existing economic growth in the developed provinces can result in a coordinated green economy.

**Figure 3:**
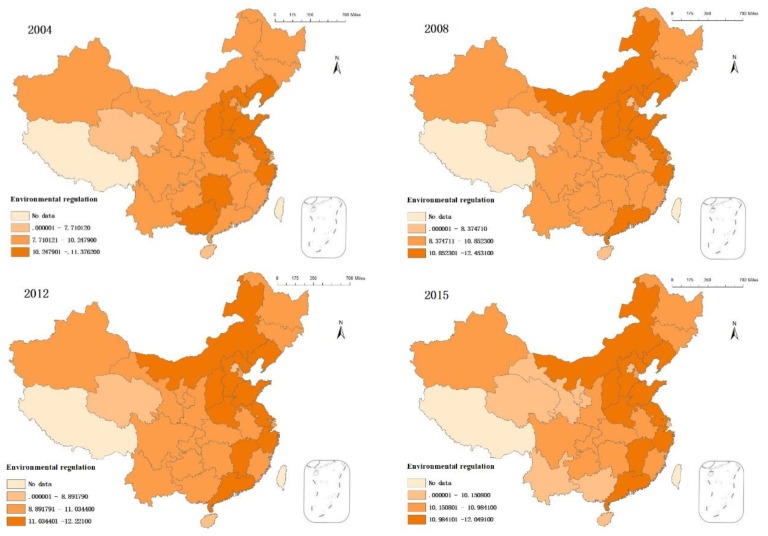
The temporal and spatial evolution of Chinese environmental regulations.

A comparison of Figure 2 and Figure 3 for the year 2004 indicates that areas with higher sewage charges had lower GTFP, while areas with lower sewage charges had relatively high GTFP. This indicates that current environmental regulations are insufficient for promoting GTFP growth. Thus, enterprises can currently continue to produce or even increase pollution emissions by paying sewage charges. In 2008, only the Guangdong Province maintained high sewage charges while maintaining third-level GTFP. This indicates that the effects of environmental regulations on green economic development have begun to appear. In 2012, the number of cities with high sewage charges did not decrease, but cities with high GTFP did. This indicates that each city maintained certain sewage charges while environmental quality was declining. This illustrates incompatibility between economic growth and environmental protection. Sewage charges in second and third-level cities decreased in 2015. Sewage charges in Guangxi, Gansu Yunnan, and Ningxia also decreased. However, GTFP was numerically higher in 2015 than it was in 2012. In 2015, environmental regulation policies gradually promoted green economic growth in some provinces.

### 4.2. Alternative Elasticity and Capital Intensity Calculations

This study calculated alternative elasticity e and capital intensity α by estimating the system equation based on relevant data from 2003 to 2015 in 30 Chinese provinces and cities. The generalized nonlinear least squares method was adopted and calculated using Stata 14.0 (StataCorp LLC, Lakeway Drive, College Station, TX, USA). Since nonlinear estimation is more sensitive to initial value settings, we used the “global optimal” method proposed by Klump et al. [34]. Alternative elasticity is the key initial value. We attempted to define various values in domain e∈(0, +∞) and find the estimation result that maximizes the log-likelihood, which is the “global optimal” estimation result. The results are shown in Table 2.

Considering the environmental constraints, most of the estimated parameter values were significant at 1%, while the substitution elasticity of capital and labor did not exceed 1. This differs from results obtained by Chen and Lian [36]. The results indicate that resource depletion and the environment impact substitution elasticity in China (the substitution elasticity in many provinces was less than 1).

The growth rate of labor efficiency $\gamma_{L}$ was less than 0 in most provinces, indicating a negative labor efficiency growth rate. The negative growth rate of labor efficiency may be because of the slow growth of labor efficiency caused by the quantity or quality of labor in these areas, which cannot meet local demand. The capital efficiency growth rate $\gamma_{k}$ was greater than 0. That is, the growth rate was positive. The growth rate of capital efficiency is a reflection of the dependence and utilization of capital in China’s economic development. The capital data used in this study was determined by finding the sum of capital stock and ecological service value. The ecological service value reflects the environmental supply capacity, while increased labor also puts pressure on the ecological environment. After ecological service value increases, capital efficiency will be greater than labor efficiency. The scale factor ξ is stable at approximately 1 and the capital intensity α is basically stable at a level slightly below 0.5.

Considering environmental constraints, the substitution elasticity in all 30 Chinese provinces and cities observed in this study exhibit complementary features. That is, an increase in capital elements will increase the marginal output of labor. This indicates that the change in element usage is less than the change in relative price under environmental constraints. Production will increase capital input with increased labor costs. Given the complementary relationship between these factors, this will increase the share of labor income and employment. In terms of numerical values, the eastern provinces and those with less environmental stress have slightly higher elasticity, while the central provinces and those in regions with relatively poor environmental conditions have less elasticity.

The capital intensity of most provinces was above 0.49. Only five provinces and cities (e.g., Xinjiang, Qinghai, Ningxia, Chongqing, and Hainan) were located between 0.48 and 0.49. Xinjiang, Qinghai, and Ningxia are geographically remote in relation to China’s developed regions. Infrastructure and capital attraction are therefore relatively weak in these areas. However, Chongqing and Hainan have capital attraction capacity. Chongqing and Hainan had higher concentrations of capital stock. However, the ecological service values in Chongqing and Hainan were small compared to other provinces, thus resulting in a capital intensity of less than 0.49. Although the capital intensity of the above five provinces and cities is less than 0.49, the difference in capital intensity between each province and city is small from a national point of view.

### 4.3. Measuring Biased Technological Progress

**Figure 4:**
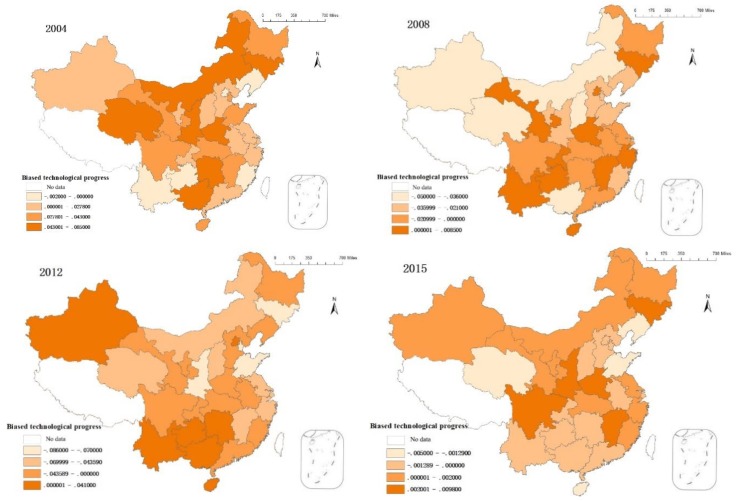
The temporal and spatial evolution of biased technological progress.

According to Equations (12)–(14), the index of technological progress in China’s 30 provinces and cities can be calculated from 2004 to 2014 using the above data (Figure 4 and Table 3).

Taking into account that 0 is the demarcation point between capital and labor bias, the index of technological progress classification was improved and divided into four levels using the ArcGIS natural discontinuous point classification method. Specific results are shown in Figure 4.

In 2004, only Beijing and Liaoning turned to labor-biased technological progress. That is, technological progress resulted in increased labor output. Other cities experienced different degrees of biased capital. In 2008, only Jiangxi, Zhejiang, and Jilin showed technological progress toward capital. Other cities have moved toward labor. From 2004 to 2008, technological progress gradually showed a trend toward labor. In 2012, the provinces achieving technological progress toward capital were Yunnan, Guizhou, Hunan, Guangxi, Hainan, and Xinjiang. Other provinces were biased toward labor. The environmental quality index has recently declined [3], and the growth of capital stocks containing environmental conditions has slowed. The use of factors has become more biased toward labor.

In 2015, only the Liaoning, Shandong, Hainan, and Qinghai provinces underwent technological progress biased toward labor (others were biased toward capital). That is, environmental quality governance increased in 2015. The growth of capital stock containing environmental qualities also increased. Factors in the national production process have turned to capital.

Table 3 indicates that China’s technological progress under environmental constraints was generally biased toward capital. Thus, technological progress is more conducive to improving the marginal output of capital. These results indicate that technological progress (whether from a labor or capital bias) was slightly smaller than that asserted by Dai and Xu [14], primarily because of environmental conditions. Environmental conditions do affect the contribution rate of technological progress to marginal capital and labor outputs. Although China has a high labor resource endowment, technological progress has generally been as biased toward capital as in developed countries. The bias of technological progress is mainly influenced by changes in factor supply. If capital accumulation increases faster than labor, enterprises tend to study the technology of biased capital, which leads to a capital bias toward technological progress [9]. The theory of transnational technology diffusion shows that technological imitation also affects the technological bias of developing countries. Developing countries typically apply the technology of capital preferences as developed by advanced countries at lower direct costs, thus affecting their technological progress in favor of capital [50].

From 2006 to 2012, the technical bias of most provinces showed a trend shifting to labor before returning to capital bias in 2013. Thus, technological progress has shifted from increasing the marginal output of labor in 2006 to increasing the marginal output of capital in 2013. Changes in factor supply are the main items affecting the bias toward technological progress. Changes in the growth rate of labor and capital have similarly significant impacts. Environmental quality seriously deteriorated after 2006 [4]. The faster one factor grows in relation to others, the more it favors technological progress. From 2006 to 2012, the capital growth rate in most provinces slowed. There was also an increase in the productivity of labor factors, which ultimately led to technological progress showing a labor bias. Therefore, the bias toward technological progress depends not only on the rate of return, but also on the scarcity of factors, the factors to which technological progress is biased, and the growth rate of relative productivity regarding those factors.

Technological progress has shown a capital bias in recent years, indicating that current capital investment has increased. The combination of technology and capital results in technological progress that gradually increases the demand for physical capital [51]. Increased labor costs and human capital have spurred capital investments, thus revealing the coexistence of economy, scarcity of labor, capital-dependent technological progress, and high labor costs. This has led to the recent development of technological progress that is biased toward capital.

### 4.4. The Spatial Effects between Biased Technology Progress, Environmental Regulations, and Green TFP

In the investigation of spatial dependence, the global Moran’s I and Geary’s c were 0.2720 and 0.4159, respectively (*z*-test values are 0.0020 and 0.0016). These results refute the original assumption at 5% significance, indicating that regional GTFP productivity has a spatial autocorrelation.

Before reunification, owing to the human capital involved in the regression, the variable may be endogenous. Hence, it was tested endogenously before the regression. This paper selects the proportion of R&D investment in each region as the instrumental variable of human capital. The Hausman test results showed that the *p*-value was 0.9471, and the null hypothesis was accepted. Thus, no endogeneity was considered.

The SDM was divided into the individual fixed-effect and the individual random effect models. We used the random effect to estimate our model according to the Hausman test results. Estimated results are shown in Table 4.

The estimation results indicate that spatial rho are positively related and that they passed the significance test. Thus, there is a significant spatial correlation between the bias of technological progress, environmental regulations, and the GTFP among provinces, given that other influencing factors do not change. As the results indicate, labor-biased technological progress can significantly increase GTFP. Biased technological progress has a greater impact on GTFP under the influence of social and economic distance weights. Companies are trying to find lower-cost production methods through economic incentive-based environmental regulations. This is expected to result in increased benefits from lower production costs. The effect of environmental regulations was negative and significant. That is, existing environmental regulations are insufficient. Most companies can reduce their environmental costs to obtain greater profits. $ER^{2}$ was positive and significant, indicating that improved environmental regulations can promote GTFP. For (1), tec×ER was significant, but tec×ER was insignificant under the influence of spatial weight. However, all were positive, indicating that the existing biased technological progress can significantly promote GTFP improvements under the influence of current environmental regulations. However, this conclusion may be limited by differences in provincial and regional development. Thus, tec×ER was insignificant under the spatial weight.

Secondary industries are primarily based on resource and energy consumption and contain negative coefficients. This indicates that it is imperative to promote the optimization and upgrade of the industrial structure to promote the conversion of new and old kinetic energy and high-quality economic growth. Economic development has radiation and spillover effects under the role of spatial weight. When the industrial scale is formed, this has certain agglomeration and scale effects. These effects enable companies to either reduce production costs or centralize pollution during the production process. Therefore, spatial secondary industry development has a positive regression coefficient for GTFP.

FDID was significant and negative under the spatial weight. This indicates that FDI includes not only foreign capital inflows, but also the transfer of a number of highly polluting and energy-intensive industries. In the face of weak environmental regulations, these shifts in pollution may make the country a “polluting paradise”. This change is more significant under the action of spatial weighting, which shows that FDID has a significant spatial spillover effect. The return of industrial structure indicates that increased current second industry is unfavorable for improving GTFP. This also indicates that current environmental regulations are insufficient for industrial development that does not coordinate with green economy growth. The coefficient of human capital stock was negative under the effect of spatial weight. This is in accordance with both the “diffusion effect” and “backflow effect” as explained by Geldahl [52]. Developed regions will attract high-tech talent inflows, thus resulting in some loss in the surrounding areas.

Technological progress and environmental regulations can no longer be individually interpreted as impacting GTFP because of the existence of spatial spillover effects. It is therefore necessary to decompose the total spatial effect to better illustrate the direct effects of technological progress on economic growth and regional spillover effects, as well as the overall effect on the entire region. The results are shown in models (3) through (5).

From the direct effect, the labor bias of technological progress will generally promote the development of GTFP. Capital reliance has slowed the growth of China’s economy, while increases in labor skills and costs has led to further improvements in the efficiency of labor use and marginal output. This will play a more direct role in promoting local and peripheral GTFP growth. At the same time, the labor bias of technology will also increase the marginal output of capital based on complementarity between these factors, thus making the use of capital more effective. The coefficient of human capital stock was highest, indicating that human capital has a significant and positive influence on GTFP. Each region attaches great importance to the role of education, thus accelerating the accumulation of human capital stock. FDID will affect GTFP to some extent. For locals, FDI inflows have significant and positive effects even though they may be accompanied by shifts to foreign energy-intensive and highly polluting industries.

Labor-biased technological progress had a significant and positive effect on GTFP under the influence of the economic spatial weight matrix. This indicates that the technical progress of labor bias reveals the “diffusion” of space. The results show that labor-force transfers in developed and peripherally undeveloped areas are accompanied by technological spillover, and that GTFP has an exemplary effect in developed areas. This will promote economic development in the surrounding area and produce a positive spillover effect.

Regarding both direct and indirect effects, ER was negative and insignificant, while $ER^{2}$ was positive and significant. However, both were significant regarding total effect. That indicates that existing environmental regulations cannot promote local or surrounding-area GTFP growth. Current environmental regulation standards cannot encourage enterprises to reduce their pollution emissions. When enterprises face this degree of environmental regulation, they continue to produce and pay increased sewage charges for the profits they can bear. However, appropriate environmental regulations may promote GTFP. Environmental regulations and GTFP are u-shaped. This indicates that existing environmental regulations are insufficient. It is therefore necessary to adjust sewage charges according to technological progress. tec×ER had a negative direct effect, but positive indirect and total effects. This indicates that technological progress under existing environmental regulations is not conducive to local GTFP growth. Regional environmental regulations are similar because of regional industrial agglomeration. The surrounding area may be affected by local environmental regulations. However, local environmental regulations are more directly effective because of differences in local government management and the implementation of local policies. This further explains the lack of regional environmental control. However, the coefficient of this interaction was not significant, indicating that technological progress has somewhat offset the inhibition effect of environmental regulations on GTFP.

As for indirect effects, the coefficient of this interaction term was positive and significant, indicating that technological progress has a significant and positive effect on the GTFP of surrounding areas under environment regulations. Technological progress itself involves spatial spillover. The transfer of labor can significantly promote GTFP growth in surrounding areas. Environmental regulation policies are currently insufficient for local green development. Technological progress can significantly promote green development in the surrounding areas under local environmental regulations because of technological spillover. Under total effect, however, this is not significant, but is subject to different regional policies. Although the labor bias of technological progress can significantly promote the development of GTFP, the role of technology is not significant under existing environmental regulations. Appropriate environmental regulations should be designed according to local conditions. Sewage standards and adjusted environmental regulations should be promptly established to promote more effective GTFP improvements under continuous technological development.

Regarding the indirect effects, the FDID coefficient was negative, but not significant. However, the direct and total effects were positive and significant. This indicates that FDI is largely conducive to China’s green economic development. Regardless, polluting industries will have negative impacts on surrounding areas. Provinces and cities thus need to create more reasonable environmental regulation policies to prevent China from becoming a foreign “pollution paradise”. Provinces and cities also need to improve their use of foreign capital while further improving the role of FDIs in green development.

The regression coefficient of the second industry was negative. The industrial structure of most provinces and cities in China is still dominated by secondary industry. This indicates that China needs to adjust its industrial structure as soon as possible while promoting optimization of the industrial structure. This will alter the influencing factors behind economic development

The regression coefficient of human capital stock was positive but not significant regarding both indirect and total effects. It is possible that this is because high-skilled labor is limited by spatial weight, and that this labor force tends to remain in areas with higher levels of economic development. The direct effect is more obvious. As a result, the regression coefficient was not significant under either indirect or total effects.

## 5. Discussion

This study has significant policy implications, outlined as follows: (1) The realization of green economic growth urgently requires changes to the impetus behind economic development. The biased development of technological progress can be altered to promote economic development. A highly skilled labor force has promoted technological progress toward a labor bias, which has become a new economic growth point regarding material capital that has slowed down economic growth. (2) In the context of China’s environmentally unfriendly economic activities, it is difficult to reduce environmental resource consumption by relying on the existing economy. Formulating a reasonable environmental control policy is the most efficient method to confront these problems.

Environmental regulation impacts are gradually emerging. We thus posit the following items: (1) Economic growth that relies on material capital is a declining trend. When the economy is moving toward a steady state, technological progress of the partial labor force will become the main impetus of economic growth. Considering the new normal conditions of economic development, China needs to optimize human capital allocation, pay attention to the accumulation of human capital, cultivate and retain talent, and increase human capital investment toward underdeveloped areas. Education and medical care investments should also be increased. This will improve the quality of education and health for citizens. China must gradually replace its “demographic dividend” with a “human capital dividend”. Thus, labor-biased technological progress mainly arises from increased human capital rather than the relative slowdown of physical capital and the environmental capital growth rate. (2) The government should formulate appropriate environmental regulations according to individual local conditions. Environmental regulation intensity is not limited to sewage charges. Such regulations should be developed according to different regions and industries; the timely adjustment and upgrade of environmental regulations and corporate emissions standards according to the tolerance of different companies will influence enterprises to actively carry out technological innovation. (3) It is reasonable to strengthen pollution controls. For instance, pollution concentration standards should be properly improved according to technological progress. Pollutants consumed by many mineral resources in China are also closely related to water pollution. Wastewater treatment should focus on coastal regions, where water resources are abundant. Conversely, solid waste and waste-gas treatments should mainly focus on areas rich in mineral resources. Air pollution issues require urgent attention and management. (4) China should continue opening-up to the outside world while actively introducing and guiding foreign investment. FDI inflows can significantly promote local and overall green economic growth. Chinese clean energy use efficiency and technology is still behind that of other developed countries. The government should therefore actively guide foreign capital allocation toward resource and energy conservation. China should also actively transform its economic development model, reasonably adjust and upgrade the industrial structure, fully optimize resource allocation, and advocate for the decisive role of the market in the optimal allocation of resources.

## 6. Conclusions

In this study, we used the “standardized supply surface system” method to estimate the direction of technological progress in various Chinese provinces, calculated GTFP through the SBM–Global–Luenberger index, and finally discussed the spatial effects of biased technological progress, environmental regulations, and green economic growth. Considering the role of technological deviation in the allocation of factors and national production, we measured the deviation of technological progress and the associated spatial effects of economic growth under environment restrictions.

Theoretical results were as follows: (1) China’s environmental constraints will slow the growth of environmental capital stock. Factor supply changes are the main items affecting the direction of technological progress. This results in technological progress that is biased toward labor. A technological bias toward labor can promote local green economic growth. However, our results revealed that technological progress was always biased toward capital. There is a complementary relationship between these elements. That is, increased capital investment can promote the marginal output of the labor factor. (2) Current environmental regulations cannot effectively promote an increase in China’s GTFP; environmental regulations and GTFP are U-shaped. However, technological progress can effectively promote peripheral GTFP growth under the influence of environmental regulations. With that said, the influence of interaction items on GTFP is not significant under direct and total effects, but the coefficient is greater than the regression coefficient of the environmental regulations themselves. Technological progress has offset environmental regulation inhibitions on GTFP to some extent. (3) Technological progress involves a spatial spillover effect. The labor bias of technological progress can currently promote this overflow. It can also promote green economic development in the surrounding area based on the effects demonstrated in developed areas. Biased technological progress enables coordinated environmental protection and economic development. The relationship between environmental regulation and GTFP was established under the influence of spatial weights, indicating that China needs to adjust its existing environmental regulation standards in time. Technological progress can only promote GTFP growth in surrounding areas under the influence of environmental regulations. The increased presence of secondary industries cannot effectively promote green economic growth while the regression coefficient of human capital stock is relatively high. China should urgently alter the industrial structure while promoting optimization and upgrading. We should simultaneously pay attention to the cultivation of talent and alter the direction of Chinese technological progress. Focus should be placed on skilled labor rather than environmental slowdowns and capital stock growth.

## Figures and Tables

**Table 1:** Summary of variables.

Models		Variable	Unit	Obs.	Mean	Std. Dev.	Min	Max
SBM–Malmquist–Global–Luenberger	Desired output	Green GDP	100 million RMB	390	6186.533	5676.814	86.1691	31,371.63
	Undesired output	So_2_	10^4^ tons	390	74.1728	44.0951	2.2	200.3
	Input	Capital stock	100 million RMB	390	10,393.95	8165.99	952.971	57,530.79
		Labor	10^4^ persons	390	4402.097	2647.705	534	10,849
		Total energy consumption	10,000 tce	390	11,711.57	7761.036	684	38,899
Spatial Durbin Model	-	GTFP	-	360	0.393	0.225	0.151	1.131093
		Educ	Natural logarithm	360	8.534	0.917	5.704	10.43347
		FDI	%	360	2.392	1.948	0.028	11.80942
		Indus	%	360	47.557	7.915	19.3	61.5
		Paiwu	Natural logarithm	360	10.551	0.994	7.354	12.53128
		Techg	-	360	0.0026	0.030	−0.36	0.128

**Table 2:** Estimated results.

Provinces	(1)	(2)	(3)	(4)	(5)	(6)	(7)
	ξ	e	α	$\gamma_{L}$	$\lambda_{L}$	$\gamma_{k}$	$\lambda_{k}$
Beijing	1.020 ***	0.999 ***	0.492 ***	−2.600 ***	0.354 ***	2.686 ***	0.362 ***
Tianjin	1.014 ***	0.947 ***	0.502 ***	−0.086 ***	1.800 ***	0.099 ***	1.494 ***
Hebei	1.012 ***	0.988 ***	0.495 ***	−0.210 **	1.151 ***	0.221 ***	1.082 ***
Shanxi	1.022 ***	0.957 ***	0.499 ***	−0.086 ***	1.555 ***	0.093 ***	1.157 ***
Inner Mongolia	1.009 ***	0.661 ***	0.498 ***	0.009 ***	0.207 ***	0.013 ***	1.298 **
Liaoning	1.008 ***	0.890 ***	0.494 ***	−0.002	4.026	0.017 ***	0.728 ***
Jilin	1.002 ***	0.653 ***	0.496 ***	0.010 ***	0.286 ***	0.003	2.254
Heilongjiang	1.016 ***	0.944 ***	0.501 ***	−0.063 ***	2.121 ***	0.082 ***	1.576 ***
Shanghai	1.010 ***	0.940 ***	0.501 ***	−0.056 ***	1.818 ***	0.065 ***	1.483 ***
Jiangsu	1.000 ***	0.719 ***	0.497 ***	0.003 *	0.202	0.009 ***	1.427 ***
Zhejiang	0.997 ***	0.691 ***	0.498 ***	0.001	0.108	0.007 ***	1.427 ***
Anhui	1.007 ***	0.839 ***	0.491 ***	0.003	0.136	0.005 **	1.274
Fujian	1.001 ***	0.901 ***	0.493 ***	−0.001	1.141	0.008 **	0.897
Jiangxi	1.001 ***	0.749 ***	0.494 ***	0.003	0.105	0.006 *	2.674
Shandong	1.003 ***	0.625 ***	0.500 ***	0.002	0.100	0.006 ***	1.160 **
Henan	1.010 ***	0.669 ***	0.493 ***	0.005 ***	0.174 ***	-0.002	3.129
Hubei	1.003 ***	0.753 ***	0.494 ***	0.008 ***	0.273 ***	0.004	1.942
Hunan	1.013 ***	0.801 ***	0.491 ***	0.011 ***	0.254 ***	0.004	1.324
Guangdong	1.020 ***	0.971 ***	0.497 ***	−0.085 ***	1.579 ***	0.092 ***	1.231 ***
Guangxi	1.005 ***	0.886 ***	0.491 ***	0.019 ***	0.221 ***	-0.001	0.002
Hainan	1.002 ***	0.948 ***	0.487 ***	0.010	0.104	-0.006	0.074
Chongqing	1.011 ***	0.957 ***	0.489 ***	0.004	0.488	0.025 ***	0.698 *
Sichuan	1.008 ***	0.798 ***	0.493 ***	0.009 ***	0.269 ***	0.007 ***	1.435
Guizhou	1.017 ***	0.998 ***	0.490 ***	0.068	0.926	-0.056	1.456
Yunnan	1.013 ***	0.997 ***	0.490 ***	0.102	1.087 ***	-0.107	1.296 ***
Shanxi	1.005 ***	0.698 ***	0.497 ***	0.007 ***	0.207 ***	0.008 ***	1.488
Gansu	1.006 ***	0.817 ***	0.492 ***	0.001	0.027	0.015 ***	0.665 **
Qinghai	1.008 ***	0.726 ***	0.485 ***	0.011 ***	0.357 ***	0.008 ***	1.097
Ningxia	1.022 ***	0.985 ***	0.486 ***	0.033	0.086 ***	−12.543	2.861 ***
Xinjiang	1.004***	0.967 ***	0.489***	0.003	0.203	0.002	0.275
Observations	13	13	13	13	13	13	13

*** *p* < 0.01, ** *p* < 0.05, * *p* < 0.1.

**Table 3:** Environmental constraints and technological progress trends in Chinese provinces with exponential distribution.

**Year**	**2004**	**2005**	**2006**	**2007**	**2008**	**2009**
D > 0	28	28	24	19	3	3
D < 0	2	2	6	11	27	27
**Year**	**2010**	**2011**	**2012**	**2013**	**2014**	**2015**
D > 0	23	20	6	27	27	27
D < 0	7	10	24	3	3	3

**Table 4:** SDM regression results.

Variables	(1)	(2)	(3)	(4)	(5)
	Main	Wx.	Direct	Indirect	Total
*tec.*	−0.169	−36.59 **	−0.251	−0.567 *	−0.818 *
	(−0.81)	(−1.97)	(−1.09)	(−1.91)	(−1.89)
ER	−0.0167	−1.212	−0.0208	−0.0211	−0.0419 *
	(−0.94)	(−1.34)	(−1.17)	(−1.43)	(−1.65)
$ER^{2}$	0.0218 ***	1.549 **	0.0264 ***	0.0266 ***	0.0530 ***
	(3.55)	(2.51)	(4.14)	(2.87)	(4.03)
tec×ER	0.371 *	14.59	−0.00167	0.00398 ***	0.00231
	(1.74)	(0.53)	(−1.31)	(2.60)	(1.06)
*indus.*	−0.00230 *	0.269 ***	−0.00423	−0.0164 **	−0.0207 *
	(−1.84)	(2.95)	(−0.74)	(−2.04)	(−1.74)
*fdid*	−0.00163	−1.015 **	0.235 ***	−0.0000829	0.235 ***
	(-0.30)	(-1.97)	(8.59)	(−0.01)	(7.13)
*educ.*	0.233 ***	−1.938 ***	0.408 *	0.251	0.659
	(8.50)	(−3.14)	(1.74)	(0.61)	(1.20)
*cons.*	−1.488 ***				
	(−5.56)				
spatial rho.	7.956 ***				
	(3.32)				
variance lgt_theta	−2.287 ***				
	(−13.02)				
sigma2_e	0.00801 ***				
	(12.49)				
Hausman	−96.43				
adjusted R^2^	0.3225				
log likelihood	372.879				

* *p* < 0.1; ** *p* < 0.05; *** *p* < 0.01.
